# Factors associated with the use of fashion braces of the Saudi Arabian Youth: application of the Health Belief Model

**DOI:** 10.1186/s12903-021-01609-w

**Published:** 2021-05-10

**Authors:** Anwar S. Alhazmi, Dania E. Al Agili, Mohammed S. Aldossary, Shaker M. Hakami, Bashaer Y. Almalki, Amal S. Alkhaldi, Mosa A. Shubayr

**Affiliations:** 1 Department of Preventive Dental Science, Faculty of Dentistry, Jazan University, Jazan, Saudi Arabia; 2 Department of Dental Public Health, Faculty of Dentistry, King Abdulaziz University, Jeddah, Saudi Arabia; 3 General Directorate of Dentistry, Therapeutic Services Deputyship, Ministry of Health, Riyadh, Saudi Arabia; 4 College of Dentistry, Jazan University, Jazan, Saudi Arabia; 5 School of Human Sciences, Faculty of Science, The University of Western Australia, 5 Stirling Highway, Crawley, 6009 WA Australia

**Keywords:** Fashion braces, Fake braces, Oral health, Health behavior, Health Belief Model

## Abstract

**Background:**

In spite of the fact that fashion braces (FBs) have been in the spotlight in recent years among both orthodontists and the general public in several countries, there is still limited evidence regarding FBs. The aim was to identify health-related behaviors in using FBs by considering the Health Belief Model (HBM).

**Methods:**

A cross-sectional study with a random sampling technique was conducted in Jazan, Saudi Arabia. The data was obtained from different middle and high schools students, and first-year students at Jazan University. The questionnaire collected data on demographic characteristics and 27 items to examine the constructs of the HBM. Chi Square test was carried out to establish the factors associated with using, the intention to use, and previous knowledge on the use of fashion braces. Logistic regression analysis was utilized to determine the factors associated with the use of fashion braces.

**Results:**

406 study participants completed the questionnaire with a response rate of 88.3%. Majority of respondents were males (52.2%) and > 19 years old (44.3%). Only 9.9% students had used the FBs. However, 25.7% of students had the intention to use them. The perceived susceptibility, perceived benefits and cues to action constructs scores were fair with the mean values of 15.38 (SD = 9.4), 28.17 (SD = 10.8) and 6.65 (SD = 2.50), respectively. However, the perceived barrier score was high with the mean values of 22.14 (SD = 7.50), and lower score of self-efficacy with mean of 8.73 (SD = 3.30). Gender, age, monthly income (family), and education level were significantly (*p* < 0.05) associated with the use of fashion braces among the study’s participants. Based on the results of logistic regression analysis family income, perceived susceptibility, and risk severity constructs predict the use of fashion braces in the study's sample.

**Conclusions:**

The current study suggests that the family income, perceived susceptibility, and risk severity of students significantly influence the use of fashion braces. Hence, it is recommended that researchers should investigate effective educational strategies and programs for improving young people’s knowledge about fashion braces and focus more on low family income students so that their using of fashion brace will be decreased.

## Background

The demand for orthodontic treatment has increased steadily over the years worldwide. Certified orthodontists recommend using orthodontic braces for teeth alignment [1]. Nonetheless, some individuals use orthodontic braces for aesthetic and fashion purposes [2]. The aesthetic value associated with fashion braces (FBs) makes them popular among teenagers [3, 4]. Unlike orthodontic braces, which are fitted by certified dentists and used for therapeutic purposes to correct problems such as under or overbites, FBs can be fitted by unauthorized street vendors, beauty salons, or the users themselves [2]. In a report by the *Times of India*, FBs are cheaper than the medically recommended braces and can cost as little as $100 [5], while braces fitted by orthodontists can cost thousands of dollars and require regular professional care over several years [6]. For example, in Thailand fashion braces can be as cheap as $45 while therapeutic braces cost $1600 or more [7].

While fashion braces have an aesthetic appeal, especially to adolescents and young adults, they pose a substantial risk to the health and well-being of users [4]. Most FBs are of inferior quality when compared to their therapeutic counterparts, and some contain lead and cadmium, which can cause poisoning, mouth damage, and cancer [3]. Using FBs raises the risk of ulceration when they are placed by non-professionals and exposed to the inner parts of the cheeks or lips. Additionally, the cement used for fixing FBs may not have been approved for oral use, thus leading to tooth decay by damaging the enamel. FBs can also lead to the unpredictable shifting of the user’s teeth, and have a negative impact on oral health-related quality of life over the short- or long-term [2, 3]. The prevalence of FBS has escalated to such a point in Thailand in recent years that authorities have banned online sales of FBs, warning they may be substandard and could even prove fatal [7]. The Dental Council in Thailand explained that authorities have banned FBs because some of these braces have leaked heavy metals, including cadmium, which can cause heart failure, liver damage, cancer, and mouth damage [3, 7].

The Bangkok Post reported that understanding the widespread use of FBs is critical, considering the adverse effects they may have on users [7]. The Health Belief Model (HBM), which emerged from social science in the 1950s, is an important tool for explaining the adoption of strategies to prevent, screen, or detect diseases early [8]. The HBM has been used widely by researchers to explain and predict health behaviors by focusing on attitudes and beliefs. In explaining its application, a study argue that people adopt health-related behaviors and actions with the hope and expectation that their actions will avert adverse health conditions [9]. The six key constructs of this model are: (1) perceived susceptibility (one’s opinion of chances of getting a condition), (2) perceived severity (one’s opinion of how serious a condition and consequences are), (3) perceived benefits (one’s belief in the usefulness of the advised action to risk or seriousness of impact), (4) perceived barriers (one’s opinion of the tangible and psychological costs of the advised action), (5) self-efficacy (confidence in one’s ability to take an action), and (6) cues to action (strategies to activate readiness to either take or not to take any action). This model is influenced and affected by several factors (modifying factors), such as age, gender, race, and ethnicity [10, 11]. The HBM also takes into account structural variables such as prior contact and knowledge about the disease, and socio-psychological variables, including social class and personality, as critical modifying factors. Studies have shown individuals are likely to adopt a recommended behavior if their perceptions about the benefits outweigh the costs and barriers [8].

When considering how to raise concerns regarding the adverse consequences of FBs with their patients, orthodontists can benefit from using the HBM. Using HBM can facilitate orthodontists in identifying the factors that either limit or facilitate the use of FBs. Several studies have explained dental and oral health behaviors using the HBM. Vaezipour et al. [12] in 2018 assessed the impact of education on the promotion of preventive dental behaviors using the HBM. Their study revealed that the provision of training on the HBM had a significant effect on knowledge, susceptibility, and self-efficacy. In their study, Buglar et al. [13] assessed the impact of self-efficacy and barriers on oral health behaviors. The study supports the hypothesis that oral health behaviors relate substantially to the enhancement of such control factors. In orthodontics, Kragt et al. [14] investigated the aspect of self-esteem in orthodontics. Their study suggests that psychological factors can also affect related oral health outcomes. Gonzalez et al. [15] investigated the psychosocial aspects of orthodontics behaviors, and they suggested that individuals’ perceptions have a substantial influence on developing a positive change to oral health. These previous studies support that the perceptions of both medical providers and patients can affect the adoption of health-related behaviors.

However, in spite of the fact that FBs have been in the spotlight in recent years among both orthodontists and the general public in several countries, there is still limited evidence in the literature regarding FBs. Encouraging self-efficacy and positive oral health behaviors, and understanding the barriers that could lead to using FBs, can help orthodontists to discourage their patients from adopting behaviours that have adverse effects on their patients and the general public. Understanding the factors that influence behaviors could enable orthodontists to engage teenagers in activities or programs to promote positive health behaviors, including knowing the dangers of FBs. This study aimed to assess and understand the health-related behavior of youth using FBs in Jazan, Saudi Arabia, and to evaluate their perceptions about this behavior, using the HBM as a crucial analytical tool. The study also examined factors that facilitate or limit the use of FBs.

## Methods

### Study design and sample size

The current study utilized a cross-sectional survey with multistage random sampling to assess and understand the health-related behavior of youth using FBs in Jazan, Saudi Arabia.According to the Saudi Ministry of Education, in 2017–2018 there were approximately 12,930 first-year students in Jazan University [16] and about 106,005 students in the region’s middle and secondary schools [17]. The sample size for the current study was calculated using an online sample size calculator (Qualtrics https://www.qualtrics.com) with a confidence level of 95% and a margin error of 5% to represent the target population. Three hundred eighty-three participants (with equal numbers of both males and females) in the region’s middle and secondary schools, as well as -first-year university schools, were included in this study. To cover any unexpected problems during the study period, an additional 20% of the original 383 participants were added to the sample. Thus, 460 participants were randomly selected, which is sufficient to address the objectives of the study.

In the first stage, four cities were selected. In the second stage, the university students were selected from Jazan city and six randomly selected middle and high schools were selected from the other three areas of the data collection. In the third stage, an admission list was obtained. Only Saudi Arabian students were included to maintain the homogeneity of the study population.

### Data collection process

A self-administrated questionnaire was conducted between October 2018 and March 2019. A list of all students and schools was obtained from the Ministry of Education and General Department of Education in Jazan region. Students from six schools (three male-only and three female-only schools) and first-year students in Jazan University were randomly selected from Jazan, Sabya, Abu Arish and Alardah cities, which is the largest cities in the region and where a large proportion of the region population resides. The inclusion criteria were that all individuals: were Saudi students aged 13 years or older, studying in middle or high school or first-year university students, and had guardian approval to participate in the study.

The questionnaire, a cover letter, and a consent form were distributed to all study participants. Students were instructed by the Principle investigator to complete or have their guardians complete the questionnaires and return them to the school administration office in 1 week. Parent of school children were asked to help if their children had diffeculty in understanding the questions. The questionnaires were collected from the students 1 week later.

Since the university students were all adults, they were asked to read the consent form and decide whether to participate or not prior to completion of the surveys. The students who agreed to participate were told how to fill in the questionnaire. The students completed the questionnaires under supervision, and the questionnaires were collected immediately after completion.

Data collection commenced after the ethics approval had been obtained from the Institutional Review Board (REC40/1-046) at the College of Dentistry of Jazan University. This was followed by requesting and receiving permission from the regional Department of Education to approach the middle and high schools. Informed consent was also taken from all potential subjects and parents of school children for participation in the current study.

### Study instrument

The HBM was used as a theoretical framework to guide the development of the questionnaire. The questionnaire was developed in English and modified from a previously validated and published questionnaire [18, 19]. The English version of the modified questionnaire was translated into the Arabic language and a back-translation method was performed to reconcile any meaningful differences between the two languages. A pre-test was conducted among 30 students (10 students from each education level) to assess the validity and reliability of the questionnaire (Cronbach α = 0.75). This questionnaire was reviewed by two dental public health professors at Jazan University to provide suggestions on clarity and accuracy. The final version of the self-administered questionnaire recorded information on demographics and the HBM constructs.

### Study variables and measures

The first part of the questionnaire included general questions about each individual’s age, gender, nationality, education level, marital status, place of residence, and family’s monthly income, as well as specific questions about FBs, such as their previous knowledge of FBs and whether they have used FBs or intend to use them. The second part included 27 items to examine the constructs of the HBM (8 questions about susceptibility and severity; 14 questions about benefits and barriers; and 5 questions about self-efficacy and cues to action) in order to gather data on the uptake of fashion orthodontics by the youth in the sample. The HBM constructs were measured using five-point semantic differential scales (1 = strongly disagree, 2 = disagree, 3 = neutral, 4 = agree, and 5 = strongly agree).

For the susceptibility and severity constructs of HBM, the respondents were asked about their beliefs regarding the behavior of using fashion braces. Perceived susceptibility to having disease was assessed by using items, e.g. “It is likely that I will get tooth decay if I use fashion braces,” “it is likely that I will get gum diseases if I use fashion braces,” and “it is likely that tooth discoloration follows if I use fashion braces.” Perceived risk of the severity of using fashion braces was assessed by items, e.g., “I would experience multiple oral ulcers if I use fashion braces,” “I would experience root resorption if I use fashion braces.” Respondents were asked about their beliefs regarding the benefits and barriers of using fashion braces. The maximum score for the susceptibility and risk severity scale was 20, which was classified into three categories: low (0–12.0 points), fair (12.1–16.0 points), and high (16.1–20.0 points).

Perceived benefits were assessed by items such as “I belief that I will not get any oral problems if I visit a qualified dentist before I use FBs.” Perceived barriers were assessed using items such as “How likely are you to get FBs if you would get negative comments from your family?” The maximum score for the perceived benefits scale was 45 scores which were classified into 3 categories: low (0–27.0 points), fair (27.1–36.0 points), and high (36.1–45 points). In addition, the maximum score for the barriers scale was 40 scores that were classified into 3 categories: low (0–24.0 points), fair (24.1–32.0 points) and high (32.1–40 points).

Self-efficacy was assessed using items such as “I feel confident that I can use FB.” Also, cues to action were assessed using three items, such as “I know where to get FBs if I need them.” The maximum score for the self-efficacy scale was 15 points, were classified into 3 categories: low (0–9.0 points), fair (9.1–12.0 points), and high (12.1–15 points). In addition, the maximum score for the cues to action scale was 10 points, which were classified into 3 categories: low (0–6.0 points), fair (6.1–8.0 points), and high (8.1–10 points). Each of the subscales was assessed separately, and the total score was calculated. All subscales with higher scores indicate stronger feelings about the construct of using fashion braces which means they are more likely to use fashion braces, except for the points assigned for barriers, which are negatively associated with the probability of using FBs.

### Data analysis

The pre-coded questionnaire was entered into IBM SPSS Statistics V25.0 to analyze the data. The scores for the HBM components were computed. Missing values and normality distribution were checked prior to analysis. Descriptive statistics (percentages, means, and number, as appropriate) were utilized to provide an overview of each variable. Chi Square test was carried out to establish factors associated with using, the intention to use, and previous knowledge of fashion braces. Logistic regression analysis was utilized to determine the factors associated with the use of fashion braces. Odds ratios and 95% confidence intervals were calculated. The significance level was set at 0.05.

## Results

Out of 460 questionnaires that were disseminated, 406 questionnaires were fully completed, which represents a response rate of 88.3%. Table 1 shows the characteristics of the study sample. The majority of respondents were males (52.2%), > 19 years old (44.3%), and non-married (88.9%). Most of the participants were from rural areas (68.0%) and 43.8% were first-year university students. Nearly 82% of the participants had an individual monthly allowance of less than 2000 SR (≈ 532 US dollars) and 49.3% had a family monthly income of less than 10,000 SR (≈ 2662 US dollars). About half (56.9%) of the participants had previous knowledge of fashion braces; 9.9% had used them, mostly purchased from the internet (42.5%); and 25.7% of the participants intended to use them.

**Table 1:** Descriptive statistics of study participants (n = 406)

Variables	N (%)
Gender	
Male	212 (52.2)
Female	194 (47.8)
Age	
13–15	69 (17.0)
16–18	157 (38.7)
>19	180 (44.3)
Marital status	
Married	20(4.9)
Not married	361(88.9)
Separated	25 (6.2)
Location	
Urban	130 (32.0)
Rural	276 (68.0)
Monthly allowance (Individual)	
0–2000	333 (82.0)
2000–4000	41 (10.1)
> 4000	32 (7.9)
Monthly income (Family)	
0–10,000	200 (49.3)
10,000–20,000	135 (33.3)
> 20,000	71 (17.5)
Education level	
Middle school	69 (17.0)
High school	159 (39.2)
1st year university	178(43.8)
Previous knowledge of fashion braces	
Yes	231 (56.9)
No	175 (43.1)
Used fashion braces	
Yes	40 (9.9)
No	366 (90.1)
Source the fashion braces	
Internet	17/40 (42.5)
Local store	10/40(25.0)
Others	13/40(32.5)
Intention to use fashion braces	
Yes	97/378 (25.7)
No	281/378 (74.3)

Table 2 shows the mean and standard deviation (SD) of each construct of the HBM framework for the use of fashion braces. The mean score (SD) for perceived susceptibility of youth students to the use of fashion braces was 15.38 (9.4). Cronbach´s alpha was 0.87 for susceptibility in this sample. This result reveals that most of the respondents had low to fair attitudes towards using FBs, as indicated by scoring on the fair level of perception with respect to the susceptibility construct. In addition, the overall arithmetic mean and standard deviation of the perceived risk severity toward using FB was 11.71 and 3.90. Cronbach’s alpha was 0.79 for risk severity in this sample. The level of overall perceived risk severity toward using FB among the participants in the study was low, since the majority of the participants had a low level of perception with respect to the severity construct. Moreover, the overall arithmetic mean and standard deviation of the variable perceived benefit was 28.17 and 10.8, respectively. Cronbach´s alpha was 0.77 for perceived benefit in this sample. The level of overall perceived benefit toward using FBs among the participants in the study was fair since the majority of the participants had low to fair levels of perceptions with respect to the perceived benefit construct. In addition, Cronbach's alpha was 0.82 for barrier to use of FBs in this sample. The overall arithmetic mean and standard deviation of the variable of perceived barrier to use was 22.14 and 7.50, respectively. This result indicated that the level of overall perceived barrier toward using FB among the participants in the study was high since the majority of the participants had a high level of perceptions with respect to the perceived benefit construct. Cronbach´s alpha was 0.79 for cues to action in this sample. The level of overall cues to action among the youth students in the study was fair. Most of the responding participants had a low to fair level of perception with respect to cues to action. Finally, Cronbach´s alpha was 0.76 for self-efficacy in this sample. The level of overall self-efficacy to use FBs among the youth students in the study was low. Most of the responding participants had a low level of perception with respect to self-efficacy.

**Table 2:** HBM Construct of Using Fashion Braces among Youth in Saudi Arabia (n = 406)

Variables	Frequency	Percentage	Mean	SD
Perceived susceptibility			15.38	9.4
Low (0–12)	220	45.2		
Fair (12.1–16)	140	34.5		
High (16.1–20)	46	11.3		
Perceived risk severity			11.71	3.90
Low (0–12)	243	59.9		
Fair (12.1–16)	123	30.3		
High (16.1–20)	46	11.3		
Perceived benefits			28.17	10.8
Low (0–27)	178	43.8		
Fair (27.1–36)	96	23.6		
High (36.1–45)	132	32.5		
Perceived barriers			22.14	7.50
Low (0–15)	14	10.1		
Fair (15.1–20)	103	25.4		
High (20.1–25)	262	64.5		
Perceived cues to action			6.65	2.50
Low (0–6)	199	49.0		
Fair (6.1–8)	96	23.6		
High (8.1–10)	111	27.3		
Perceived self-efficacy			8.73	3.30
Low (0–9)	247	60.8		
Fair (9.1–12)	102	25.1		
High (12.1–15)	57	14.0		

*HBM* Health Belief Model, *SD* standard deviation

Chi-Square was carried out determine if there are significant difference in mean the history of using fashion braces based on gender, age, marital status, location, monthly allowance (individual), monthly income (family), and education level. The results of the analysis are presented in Table 3. Gender, age, monthly income (family), and education level were significantly (*p* < 0.05) associated with the use of fashion braces among the study’s participants. Male students, students who were 19 years or older and are in their first year of university studies, students enrolled in the first year of university, and students whose monthly income was less than 10,000 SR were more likely to use FBs than their respective counterparts. In addition, the results showed that there is no significant association between intention to use FBs and the selected variables. Age, marital status, monthly income (family), and education level were significantly (*p* < 0.05) associated with participants having previous knowledge of fashion braces. The results showed that students who were 19 years or older and studying in their first year at university, unmarried students, and students with monthly income between 10,000 and 20,000 SR were more likely to use FBs than their respective counterparts.

**Table 3:** The mean HBM model structure with history of using fashion braces

Variables	Use FBs			Intend to Use FBs			Previous knowledge of FBs		
	N (%)		*p* value	N (%)		*p* value	N (%)		*p* value
	Yes	No		Yes	No		Yes	No	
Gender									
Male	27(6.7)	185(45.5)	**0.03**	44(11.6)	150(39.7)	0.11	121(29.8)	91(22.4)	0.51
Female	13(3.2)	181(44.6)		53(14.0)	131(34.7)		110(27.1)	84(20.7)	
Age									
13–15	7(1.7)	62(15.3)	**0.02**	19(5.0)	50(13.2)	0.74	41(10.1)	28(6.9)	**0.00**
16–18	8(2.0)	149(36.7)		32(8.5)	105(27.8)		70(17.2)	87(21.4)	
> 19	25(6.2)	155(38.1)		46(12.2)	126(33.3)		120(29.6)	60(14.8)	
Marital status									
Married	3(0.7)	17(4.2)	0.18	5(1.3)	15(4.0)	0.90	10(2.5)	10(2.5)	**0.03**
Not married	37(9.1)	324(79.8)		87(23.0)	248(65.6)		213(52.5)	148(36.5)	
Separated	0(0.0)	25(6.2)		5(1.3)	18(4.8)		8(2.0)	17(4.2)	
Location									
Urban	13(3.2)	117(28.8)	0.54	29(7.7)	90(23.8)	0.40	72(17.7)	58(14.3)	0.38
Rural	27(6.7)	249(61.3)		68(18.0)	191(50.5)		159(39.2)	117(28.8)	
Monthly allowance (Individual)									
0–2000	35(8.6)	298(73.4)	0.63	82(21.7)	229(60.6)	0.77	194(47.8)	139(34.2)	0.28
2000–4000	3(0.7)	38(9.4)		9(2.4)	29(7.7)		23(5.7)	18(4.4)	
> 4000	2(0.5)	30(7.4)		6(1.6)	23(6.1)		14(3.4)	18(4.4)	
Monthly income (Family)									
0–1000	17(4.2)	189(46.6)	**0.009**	48(12.7)	137(36.2)	0.53	90(22.2)	110(27.1)	**0.00**
10,000–20,000	12(3.0)	118(29.1)		29(7.7)	98(25.9)		94(23.2)	41(10.1)	
> 20,000	11(2.7)	59(14.5)		20(5.3)	46(12.2)		47(11.6)	24(5.9)	
Education level									
Middle school	7(1.7)	62(15.3)	**0.02**	19(5.0)	50(13.2)	0.80	41(10.1)	28(6.9)	**0.00**
High school	8(2.0)	151(37.2)		33(8.7)	106(28.0)		72(17.7)	87(21.4)	
1st year university	25(6.2)	153(37.7)		45(11.9)	125(33.1)		118(29.1)	60(14.8)	

Chi square test used to test for significance

Bold font indicates statistical significance

*SD* Standard deviation

A logistic regression was performed to ascertain the effects of gender, age, location, monthly income (family), having previous knowledge of FB, and the HBM constructs on the likelihood that participants will use fashion braces (Table 4). The model explained 19.9% (Nagelkerke *R*^2^) of the variance in using fashion braces and correctly predicted 90.6% of participants. The family monthly income was associated with the likelihood of fashion braces use among the youth in our study. Adjusted for all other factors, the odds of using fashion braces was about 4.19 times higher among participants whose family’s income was less than 10.000 SR (OR 4.19; 95% CI 1.61–10.89) and 2.09 times higher among those living in families with an income between 10,000 and 20,000 SR (OR 2.09; 95% CI 1.06–7.14), compared to those living in families with incomes greater than 20,000 SR. The study also shows that perceived susceptibility and severity of the HBM constructs predicted the use of fashion braces. The study found that, as perceived susceptibility increases, the odds of using FBs increase approximately 1.25 times (OR 1.25; 95% CI 1.08–1.45). Lastly, as perceived risk severity increases, the odds of using FB increases approximately two times (OR 1.81; 95% CI 0.70–0.94).

**Table 4:** Logistic regression model of the factors associated with using fashion braces

Variables	Parameter estimate	OR	95% CI
Gender			
Male	− 0.272	0.76	(0.34–1.71)
Female			
Age			
13–15	0.059	1.06	(0.39–2.86)
16–18	0.714	2.04	(0.80–5.23)
> 19	[Reference]		
Location			
Urban	0.167	1.18	(0.55–2.53)
Rural	[Reference]		
Monthly income (Family)			
0–10,000	1.43	4.19	**(1.61–10.89)**
10,000–20,000	0.734	2.08	
> 20,000	[Reference]		**(1.06–7.14)**
Previous knowledge of fashion braces			
Yes	− 0.573	0.56	(0.23–1.38)
No	[Reference]		
Susceptibility	0.223	1.25	**(1.08–1.45)**
Risk severity	0.213	1.81	**(0.70–0.94)**
Benefits to action	0.047	1.05	(0.99–1.10)
Barriers to action	0.008	1.01	(0.95–1.07)
Cues to action	− 0.098	0.91	(0.76–1.08)
Self-efficacy	0.013	1.01	(0.89–1.16)

R^2^ = 0.199; percentage correct = 90.6%

Bold font indicates statistical significance

*OR* odd ratio, CI confidence interval

## Discussion

The aim of this study was to assess the behaviour of using fashion braces among Saudi youths in the Jazan region using the HBM in order to evaluate the level of perception about this behaviour among middle school, high school, and first-year university students. The study also examined factors that facilitate or limit the use of fashion braces. The current study is the first to apply the HBM to determine the factors that influence the use of fashion braces in Saudi Arabia. Due to lack of data from previous studies in this area, some findings were not comparable.

The findings were used to identify the beliefs of students regarding the use of fashion braces. Only a few (9.9%) students reported using fashion braces, which was higher when compared with a previous study where 3.4% of participants used FB [2]. Among those who did not use fashion braces, 25.7% intend to use them. This indicated the need for further investigation about the reasons of using fashion braces. This finding aligns with the result of only a few participants acknowledging that the use of fashion braces is associated with oral disease and adverse oral health consequences. Students were likely misinformed about the health issues and adverse health consequences that come from using fashion braces and this might because they likely had not consulted with orthodontists.

In the present study, the majority of participants considered themselves to be at low risk of developing oral disease if they use FBs. This is confirmed with the result that over 59% of the participants had a low level of perception to the statements related to the seriousness of the disease. This indicated that many of them might be confused or had low knowledge on the potential severe consequences of using FBs [4]. This also aligns with the result in this study that almost a third of the participants believed that using FBs would help them to have psychological benefits such as a good appearance. This is consistent with findings that people received more psychological benefits by using FBs, although they could experience consequences such as pronunciation and diet problems [2]. However, many respondents (60.8%) were not confident in their ability to use FBs. This apparent discrepancy may be attributed to the low awareness of using FBs among the participants, and because the use of FBs could be a shared decision with the participants' families.

This study found that monthly income of the family and perceived susceptibility and risk severity were significant predictors of the use of FBs after controlling for other factors. Clearly, having a high level of perceived susceptibility and risk severity, low family income will increase the use of fashion braces. Students whose families have incomes less than 10,000 SR were more likely to report using fashion braces compared to those with higher income. This could be because those families could not afford the cost of therapeutic braces, making the price of fashion braces more affordable for their needs. This result is supported by studies which found that financial considerations were one of the main reasons for patient treatment decisions and their demand for dental braces [6, 20]. The high level of perceived susceptibility and risk severity could be because of the lack of knowledge regarding FBs, which has also been reported in different studies as a reason for using FBs [3, 4]. Mostly the participants in previous studies thought that FBs can provide a faster and cheaper treatment when compared to the therapeutic orthodontic appliances.

There were several strengths of this study. This study was a cross-sectional study that allowed researchers to capture a snapshot of the target population regarding the fashion braces. Cross-sectional studies provide a quick and inexpensive method of collecting useful information [21]. This study is particularly useful in informing the planning of education programs in schools regarding the use of fashion braces and the consequences of doing so. There are several limitations that should be considered when utilizing the results of this study. First, our data are cross-sectional and, hence, can be interpreted only as an association rather than a cause-effect relationship. In addition, the study was conducted among students from only four cities in the Jazan region; hence, the findings of the study may not be generalizable to students living in other cities in Jazan or in Saudi Arabia in general. Researchers should investigate effective educational strategies and programs for improving young people’s knowledge about fashion braces, as the current study clearly shows that a relatively large proportion of Saudi students use fashion braces or have the desire to use them. These education programs should focus more on students with low family income.

## Conclusion

The current study is the first to apply the Health Belief Model to determine the factors that influence the use of fashion braces among Saudi students in the Jazan region. Family income, perceived susceptibility, and risk severity significantly influence the use of fashion braces. Further research focusing on different cities or geographical regions in the KSA is needed to validate this finding. Finally, the findings of this study support the need for more educational programs in middle and high schools and universities, which are needed to clarify the consequences of using fashion braces.

## Data Availability

The datasets used and/or analysed during the current study are available from the corresponding author on reasonable request.
